# Supplementary material for: Expanding Phenotype of Poirier–Bienvenu Syndrome: New Evidence from an Italian Multicentrical Cohort of Patients
Source: Genes (Basel). 2022 Jan 30;13(2):276. doi: 10.3390/genes13020276 (PMC8872204; doi:10.3390/genes13020276)
Supplement: Supplementary file 1 [file genes-13-00276-s001.zip › genes-1561166-supplementary.pdf]

**Manuscript Title**: Expanding Phenotype of Poirier-Bienvenu Syndrome: New Evidences From An Italian Multicentrical Cohort of Patients

**Manuscript ID:** genes-1561166

**Authors**: Orsini A, Santangelo A, Bravin F, Bonuccelli A, Peroni D, Battini R, Foiadelli T,Bertini V, Valetto A, Iacomino M, Nigro V, Torella A, Scala M, Capra V, Vari MS, Fetta A, Di Pisa V, Montanari F, Epifanio R, Bonanni P, Giorda R, Operto FF, Pastorino G, Singacili E, Okuyaz C, Bozdogan S, Musante L, Faletra F, Zanus C, Ferretti A, Vigevano F, Striano P, Cordelli DM

**Journal:** Genes

Hereby we provide the form used to collect data from each centre.

| | | |
|---|---|---|
| Centre | | |
| Patient ID | | |
| Birth date | | |
| Sex | | |
| GENETIC MUTATION | | |
| Variant and Exon involved | Variant | Exon |
| Type of mutation (e.g.: nonsense, missense, frameshift…) | | |
| Genetic analysis performed | | |
| EPILEPSY | | |
| Epilepsy onset (months) | | |
| Epilepsy type | | |
| Further epilepsy types showed | | |
| DISABILITY | | |
| Intellectual disability (specify scale adopted) | | |
| Motor skills | | |
| Hypotonia | | |

| | |
|---|---|
| Dystonia | |
| Speech | |
| Communication disability | |
| Learning disability | |
| **IMAGING AND EEG** | |
| EEG at onset (if available) | |
| EEG during follow-up | |
| MRI | |
| Other imaging (if available) | |
| **OTHER FEATURES** | |
| Facial Dysmorphism | |
| Micro-/Macrocephaly (yes/no) | |
| Autistic features | |
| Immunological features | |
| Endocrinological features | |
| Cardiology features | |
| Gastrointestinal features | |

| | |
|---|---|
| Orthopedic features | |
| ENT features | |
| Other abnormalities | |
